# Transcriptomic Determinants of Scrapie Prion Propagation in Cultured Ovine Microglia

**DOI:** 10.1371/journal.pone.0147727

**Published:** 2016-01-25

**Authors:** Juan F. Muñoz-Gutiérrez, Sebastián Aguilar Pierlé, David A. Schneider, Timothy V. Baszler, James B. Stanton

**Affiliations:** 1 Department of Microbiology and Pathology, College of Veterinary Medicine, Washington State University, Pullman, Washington, United States of America; 2 United States Department of Agriculture, Agricultural Research Service, Pullman, Washington, United States of America; 3 Department of Pathology, College of Veterinary Medicine, University of Georgia, Athens, Georgia, United States of America; University of Maryland School of Medicine, UNITED STATES

## Abstract

Susceptibility to infection by prions is highly dependent on the amino acid sequence and host expression of the cellular prion protein (PrP^C^); however, cellular expression of a genetically susceptible PrP^C^ is insufficient. As an example, it has been shown in cultured cells that permissive and resistant sublines derived from the same parental population often have similar expression levels of PrP^C^. Thus, additional cellular factors must influence susceptibility to prion infection. The aim of this study was to elucidate the factors associated with relative permissiveness and resistance to scrapie prions in cultured cells derived from a naturally affected species. Two closely related ovine microglia clones with different prion susceptibility, but no detectable differences in PrP^C^ expression levels, were inoculated with either scrapie-positive or scrapie-negative sheep brainstem homogenates. Five passages post-inoculation, the transcriptional profiles of mock and infected clones were sequenced using Illumina technology. Comparative transcriptional analyses identified twenty-two differentially transcribed genes, most of which were upregulated in poorly permissive microglia. This included genes encoding for selenoprotein P, endolysosomal proteases, and proteins involved in extracellular matrix remodeling. Furthermore, in highly permissive microglia, transforming growth factor β–induced, retinoic acid receptor response 1, and phosphoserine aminotranspherase 1 gene transcripts were upregulated. Gene Set Enrichment Analysis identified proteolysis, translation, and mitosis as the most affected pathways and supported the upregulation trend of several genes encoding for intracellular proteases and ribosomal proteins in poorly permissive microglia. This study identifies new genes potentially involved in scrapie prion propagation, corroborates results from other studies, and extends those results into another cell culture model.

## Introduction

Transmissible spongiform encephalopathies (TSEs, a.k.a., prion diseases) are a group of lethal and incurable neurodegenerative diseases caused by prions. TSEs include Creutzfeldt-Jakob disease (CJD) in humans, bovine spongiform encephalopathy in cattle, scrapie in sheep and goats, chronic wasting disease in cervids, and others [1]. Prions are primarily, if not solely, composed of PrP^D^ (D superscript for “disease associated”), which is a misfolded isoform of the cellular prion protein (PrP^C^) [2]. Conversion of PrP^C^ into PrP^D^ is the central event in the pathogenesis of TSEs, and according to the protein-only hypothesis [2] PrP^D^ catalyzes the conversion of PrP^C^ into a likeness of itself by an incompletely understood mechanism of replication. Unlike PrP^C^, PrP^D^ is primarily composed of β–pleated sheets; this abnormal conformational state is transmitted to newly converted molecules of PrP^D^ and is associated with distinct biochemical features: aggregation, detergent insolubility, and partial proteinase K resistance [1].

A major determinant of a host’s susceptibility to prion infection and replication is the sequence identity between the host’s PrP^C^ with that of the infectious PrP^D^. For instance, the naturally occurring Q171R and E219K variants of PrP^C^ are known to render sheep and humans resistant to classical scrapie and CJD prions, respectively [3, 4]. Moreover, PrP^C^ expression is required for prion propagation in cultured cells [5]. However, the inability of some PrP^C^ expressing cell lines to propagate prions [6, 7] indicates that additional factors must play a role in susceptibility to prion infection. Identification of such factors would greatly improve the understanding of TSEs pathogenesis and enable identification of much-needed therapeutic targets.

Previous studies have identified genes potentially involved in the pathogenesis of TSEs, but have shortcomings with respect to the techniques and models employed. For instance, microarray technology has been used on ovine (natural TSE host) [8, 9] and murine (adapted TSE host) [10, 11] tissues to identify the transcriptional responses during prion infection. However, these studies could not differentiate if the transcriptional differences were directly related to prion propagation or were simply secondary to infection, nor could they assign gene transcription status to specific cell types. Additional attempts to define the factors that affect cellular permissiveness to prion infection include transcriptomic analyses through microarray technology on cultured cells [12–14]. The results of the latter studies have provided new insights into prion infection susceptibility in a pathophysiologically relevant cell type (i.e., neurons); including the role of proteins associated with extracellular matrix remodeling [13] and others that may alter trafficking of PrP^C^ and PrP^D^ [12] during prion propagation. Due to the difficulty in translating the biological relevance of results from cell cultures to whole organisms, and the technical limitations of microarray technology [15, 16], the aforementioned studies would be complemented by comparing the transcriptional profiles of another pathophysiologically relevant cell type using high-throughput RNA-Seq. This technique would allow for transcriptomic analysis with a broader dynamic range and a greater ability to detect low abundance novel transcripts.

We have established an immortalized ovine microglia cell culture system permissive to natural scrapie isolates (i.e., derived directly from brainstems of sheep infected with classical scrapie) [7]. Microglia are myeloid-derived monocyte cells that function in the central nervous system as resident macrophages and which have been shown to have important roles in the transport [17], accumulation [18], and degradation of prions [19] in the central nervous system and peripherally within the body. Furthermore, microglia contribute to the maintenance and degradation of the CNS extracellular matrix [20]. Similar to murine and rabbit model cell culture systems used for TSE research [6, 21], only a relatively small proportion of immortalized microglia sublines were found to be permissive to prions [7]. Also, PrP^C^ expression levels failed to predict susceptibility to either natural scrapie isolates or culture-adapted prions across multiple ovine microglia sublines. This indicates that, in this cell line, susceptibility to scrapie prions is determined by additional factors and not only by PrP^C^ levels. In the present study, the transcriptional profiles of highly permissive and poorly permissive ovine microglia clones were compared using RNA-Seq to test the hypothesis that a distinct transcriptional signature is associated with prion susceptibility in cultured ovine microglia. Herein, twenty-two genes with consistent differential transcription between highly permissive and poorly permissive ovine microglia cells are identified and their hypothetical roles in prion propagation at the cellular level are discussed. As such, this is the first comprehensive comparative transcriptional study that characterizes prion susceptibility in cultured cells using high throughput RNA sequencing technology.

## Materials and Methods

### Cell culture and inoculation with natural scrapie prions

Previously established hTERT-microglia cells [7] were used for this study. Subline H cells from a previous study [7] were cloned by limiting dilution to generate clones 438 and 439 and these were previously characterized as monocyte-derived cells by expression of CD14 [7]. Cryogenically stored clones 438 and 439 were thawed and maintained in Opti-MEM medium supplemented with 10% heat-inactivated fetal bovine serum (Atlanta Biologicals) 2 mM L-glutamine, 10 IU of penicillin, and 10 mg/ml streptomycin. Previously, in a single experiment, it was demonstrated that these clones have differential permissiveness to a scrapie isolate derived from a naturally infected sheep [7]. This phenotypic difference in scrapie-permissiveness was confirmed for the current study by challenging microglia clones with scrapie-positive and scrapie-negative brainstem homogenates. For inoculation, cells were plated at a concentration of 4 x 10^5^ cells/well in 12-well plates and inoculated with 1% (w/v) brainstem homogenates (for additional information on inoculum preparation, inoculation protocol, and sources see [7]). Inocula made from the X124 natural scrapie isolate [22] and from a genotype-matched, scrapie-naïve lamb were respectively used as scrapie-positive and scrapie-negative controls. Inoculum was replaced with fresh culture medium seven days after inoculation and cells were kept in culture for one week prior to expansion to 25-cm^2^ flasks. Then, cells were split 1/5 every seven days. A total of three inoculation experiments were performed, each including three culture replicates. Permissiveness to scrapie prion infection was defined as the accumulation of nascent PrP^Sc^ (Sc superscript for scrapie) at the third and fourth passage post-inoculation. PrP^Sc^ was assessed by immunoblotting and ELISA.

### Detection of PK-resistant PrP with immunoblotting

At the third passage post-inoculation, cells were collected and lysed for immunoblotting as previously described [7]. In brief, cell lysates were first normalized to total protein using the bicinchoninic acid protein assay kit (Thermo Scientific) before digestion with 50 μg/ml PK (Roche) for 1 h at 37C immediately before immunoblotting. In some experiments (S1 Fig), to increase the sensitivity of this assay, phosphotungstic acid (PTA) was used to precipitate PK-resistant PrP prior to immunoblotting, as previously described [23]. The anti-PrP monoclonal antibody 99/97.6.1 (which detects a conserved residue on the C-terminus of PrP [24]) was used for immunoblotting at a concentration of 3.5 μg/ml. Immunoblots were visually interpreted as either positive or negative. Samples that were not treated with PK were included as controls for protein extraction and PrP detection.

### Detection of misfolded PrP with ELISA

**Fig 1:**
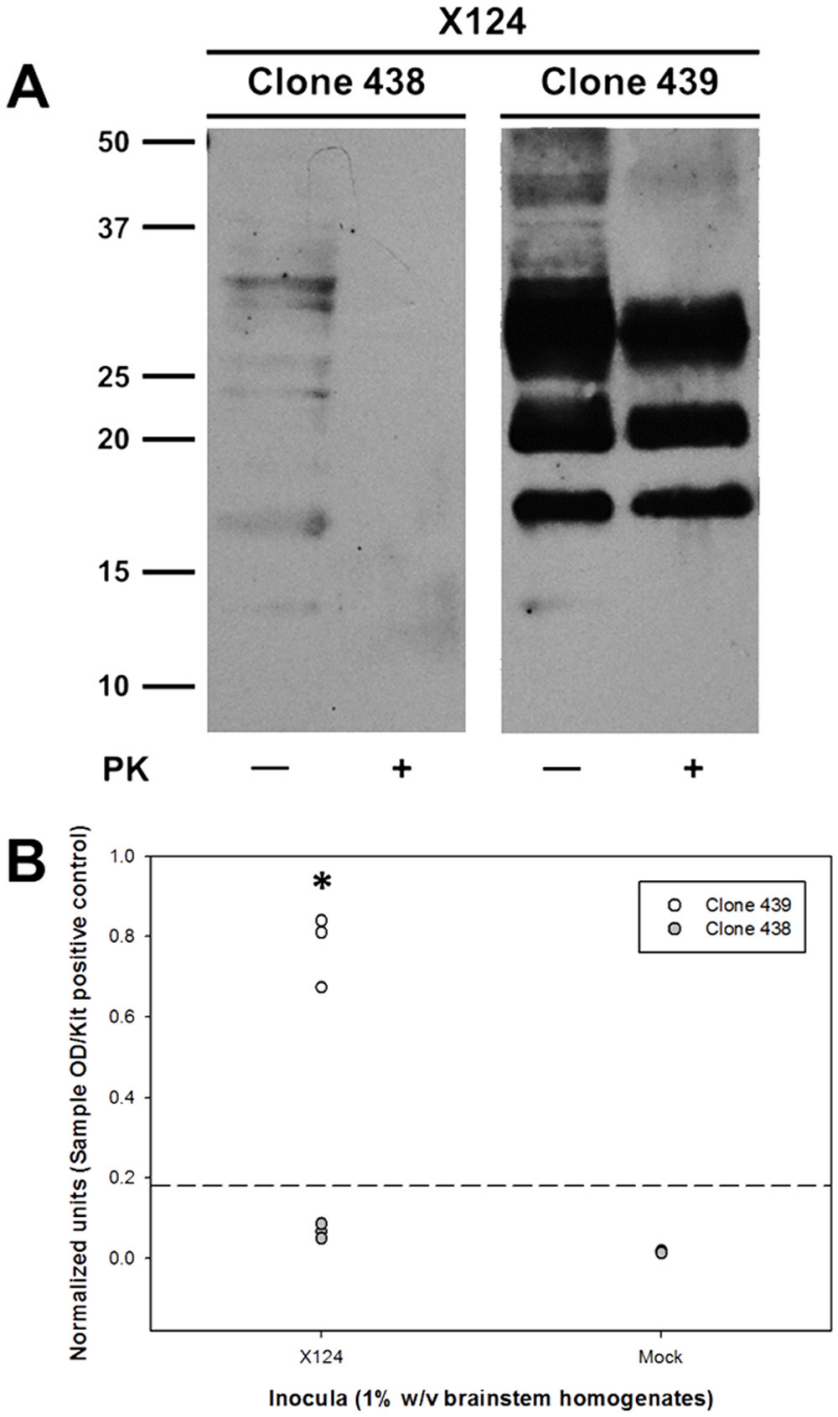
Characterization of differential prion susceptibility in ovine microglia clones. Microglia clones were inoculated with 1% (w/v) brainstem homogenates from either scrapie-positive (“X124”) or scrapie-naïve (“Mock”) sheep. Inoculated cells were passaged on a weekly basis and then tested for the accumulation of nascent PrP^Sc^ at passage three by immunoblotting (A) and at passage four by ELISA (B). Immunoblot picture (A) depicts results from one culture replicate inoculated with scrapie-positive brain homogenates and is representative of three independent experiments. In graph (B), each circle represents the mean of three culture replicates from each of three independent experiments (i.e., total of six circles per treatment, three for 439 and three for 438), and the dashed line indicates the assay cut-off threshold for detection of PrP^Sc^. Values of normalized units in the X124 group for clone 439 are significantly higher than those of in clone 438 (*: *P* = 0.0048, unpaired *t*-test). Values of normalized units between X124 and mock groups are statistically significantly different for the clone 439 (*P* = 0.0044, paired *t*-test) and clone 438 (*P* = 0.0065, paired *t*-test); however, the values for clone 438 fail to rise above the cut-off threshold and are negative by immunoblotting ([A] and S1 Fig).

At the fourth passage post-inoculation, cells were collected and lysed for detection of PrP^Sc^ using the Herd-Check CWD Ag Test (IDEXX) as previously described [23]. The corrected optical density (OD) values (OD_450_–OD_620_) of each experiment were each divided by that experiment’s kit-provided positive control to normalize for ELISA plate-to-plate variation; thus, the combined experiment results in Fig 1B are reported as “normalized units”. The kit-provided cut-off value (which is the average of three kit-provided negative controls + 0.18) was used as threshold to determine accumulation of PrP^Sc^. In addition, the corrected OD values of lysates from scrapie-inoculated cells from clone 438 were compared to those of clone 439 using an unpaired *t*-test with a level of significance of *P* < 0.01. Also, the corrected OD values of lysates from scrapie-inoculated cells were compared to those of mock-inoculated cells from each clone using a paired *t*-test with a level of significance of *P* < 0.01. Statistical analyses were done using JMP Pro 11.2.0.

### PrP^C^ quantification

The levels of cell-associated PrP^C^ of each uninoculated ovine microglia clone were determined using a commercial ELISA (TeSeE^™^ SAP Detection Kit, Bio-Rad) following manufacturer’s instructions, as previously described [7]. This kit uses an immobilized anti-PrP antibody for plate capture, but enzymatic digestion using PK was not utilized (i.e., the TeSeE^™^ SAP Purification Kit was not used) so that total PrP (i.e., PrP^C^ only in uninoculated cells) content could be measured. Briefly, cell lysates were normalized to total protein with the bicinchoninic acid protein assay kit (Thermo Scientific) and the appropriate dilutions of cell lysates were added to the ELISA plate. A PrP^C^ standard curve was prepared using half-log dilutions of uninoculated sheep PrP^C^ expressing RK-13 (Rov) cell lysates to convert corrected OD values to relative amounts of PrP^C^, as previously described [7]. Lysates from three technical replicates, each with three culture replicates, of each ovine microglia clone were analyzed. The kit-provided positive and negative controls were included in all the assays. The levels of PrP^C^ between highly permissive and poorly permissive clones were compared with an unpaired *t*-test with a level of significance of *P* < 0.05 using JMP Pro 11.2.0.

### RNA isolation and sequencing

At the fifth passage after inoculation, total RNA was extracted from three culture replicates of each ovine microglia clone. TRIzol (Invitrogen) was used to lyse cells in culture flasks following manufacturer’s instructions. Total RNA was analyzed with the 2100 Bioanalyzer (Agilent Technologies) and enriched poly(A) RNA (selected with the PrepX PolyA mRNA isolation kit [Wafergen biosystems]) was used to prepare cDNA libraries. Samples were sequenced using Illumina HiSeq 2000 with 100 bp reads and all were run on a single lane. The raw sequence data have been deposited in the NCBI Sequence Read Archive (accession number: PRJNA257519).

### Comparative transcriptional analysis

The CLC Genomics Workbench (CLC Bio.) was used to process RNA-Seq data. Mapping parameters were adjusted to map a maximum number of reads to the reference *Ovis aries* Oar_v3.1 [25]. The distribution of the expression values for all samples was analyzed and compared. Normalization by quantiles was applied to adjust the distributions for further comparison, as previously described [26]. Fold changes with respect to RPKM (Reads Per Kilobase per Million mapped reads) values were calculated. Baggerley’s statistical test on proportions was applied to evaluate significance of fold changes [27]. Bonferroni correction was used to minimize the occurrence of false positives. Comparisons of replicates were performed in order to account for variation of transcription values within each experimental group. Genes with Bonferroni corrected *P* values below 0.05 were selected for further evaluation.

### Evaluation of genes encoding for hypothetical proteins

The available translated sequences of genes encoding hypothetical proteins were analyzed for conserved domains using the NCBI’s conserved domain database [28]. In a few cases, translated sequences were generated with the Translate tool of the ExPASy Bioinformatics Resource Portal [29] prior to domain identification. Domains with the lowest Expect value (E-value) were considered for further evaluation.

### K—means clustering

K—means clustering was used to assign the mean transcriptional value of each gene to the cluster whose center is nearest, as previously described [26]. Euclidean distance was used as the distance metric and five partitions were used to generate the clusters. The mean gene expression value over all input samples was subtracted from all genes. Previously normalized expression values were used for clustering.

### Gene Set Enrichment Analysis

In order to evaluate different GO (Gene Ontology) biological pathways, the Gene Set Enrichment Analysis (GSEA) test [30] was used as previously described [26]. The test calculates and uses ANOVA statistic for multiple group experiments for each feature, as measures of association. The *O*. *aries* genome was annotated using the *Bos taurus* GO annotations [31]. Briefly, feature IDs in the *O*. *aries* genome were matched to synonyms and gene products from IDs of the *Bos taurus* data base. Additionally, this was complemented with manual curation of the annotation file, including genes reported in the Kyoto Encyclopedia of Gene and Genomes (KEGG) [32].

### Quantitative RT-PCR

RT-qPCR was used to confirm RNA-Seq results of selected genes employing the SsoAdvanced^™^ Universal SYBR Green Supermix (Bio-Rad). In brief, total RNA was collected from cells inoculated with scrapie-positive homogenates (nine culture replicates total per each microglia clone) at the sixth passage post-inoculation using the RNeasy Mini Kit (QIAGEN) following manufacturer’s instructions. Approximately 1 μg of total RNA was reverse-transcribed using the SuperScript^®^ III First-Strand Synthesis SuperMix for RT-qPCR (Life Technologies) and 2 μl of cDNA were used for qPCR in a 20-μl reaction. Nine genes with differential transcription based on RNA-Seq analysis were analyzed by RT-qPCR, and these genes were selected based on their consistent up- or down-regulation across pair-wise comparisons. Reaction conditions for RT-qPCR were 95C for 30 s, 35 cycles of denaturation at 95C for 15 s and annealing at 60C for 10 s followed immediately by a melt curve. Negative controls for RT-qPCR included no-template controls. A standard curve was used for each run to calculate the amplification efficiency of each run and all calculations of relative expression were based on experiments with ≥ 95% efficiency. The constitutively expressed *GAPDH* gene was used for normalization. Also, the *PRNP* gene was analyzed to support ELISA results regarding PrP^C^ quantification. Primers used in this study are shown in Table 1 and all but those for *PRNP* [33] and *GAPDH* [34] were designed using the primer-BLAST tool [35] based on the reference *O*. *aries* Oar_v3.1 [25]. Each primer set was assessed through gradient PCR to determine the optimum annealing temperature and PCR products were analyzed through gel electrophoresis to confirm the size was of the expected molecular weight. The ratios of relative expression were calculated through the REST software [36], which uses a mathematical model that includes the efficiency correction of individual transcripts and tests the expression ratio results of the investigated transcripts for significance by a randomization test.

**Table 1:** Primers used for RT-qPCR.

Target gene	Forward primer sequence	Reverse primer sequence	Amplicon size (bp)
*CTSB*	TTGGAAGGCTGGACACAACT	TCCCTGGTCTCGGATCTCTT	190
*DCN*	GCTGGCCGACCATAAGTACA	TGGGTTGCTGAAAAGGCTCA	139
*DPT*	GCTGGTGGGAGGAGATCAAC	GACTCGAAGTAGCGGCTCTG	97
*MMP14*	CGCTATGCCATCCAGGGACT	CTCCCACACTCGGAATGCCT	126
*PRNP* [33]	CCGTTACCCCAACCAAGTGT	CGCTCCATTATCTTGATGTCAGT	159
*PTN*	GCAGACTCCACAGTACCTGC	ACACACACTCCACTGCCATT	163
*RARRES1*	GGCAGCTCTTACGTGATGTG	CCAGACCAAGTGAATACGGCA	177
*SEPP1*	ACCGTGGTTGCTCTTCTTCAA	TCTCCAGTTTTACTCGCAGGTC	85
*SQSTM1*	TTGTACCCACATCTGCCACC	AGCCGCCTTCATCAGAGAAC	91
*TGFBI*	TGGGCGGCAAGAAACTGAGA	GCGATTGTCCCCCTTCAGGA	170
*GAPDH* [34]	GGCGTGAACCACGAGAAGTATAA	CCCTCCACGATGCCAAAGT	120

### Identification of genes enriched in ramified and amoeboid microglia

The RNA-Seq database generated from the above experiments was used to further characterize the cell type. Given the lack of specific information of such genes in domestic sheep, a database from rats [37] was used as reference for genes enriched in ramified (42 genes) and amoeboid (43 genes) microglia. The ≥1 RPKM criterion proposed by Hebenstreit et al [38] was used as the cut-off for genes being actively transcribed (i.e., “present”).

## Results

### Prion permissiveness in ovine microglia clones

To limit the extent of phenotypic and genetic heterogeneity, two first-generation clones (clones 438 and 439 [7]), which were derived from the same subline (subline H [7]) but have differential prion permissiveness, were selected. Cells were inoculated with scrapie-positive and scrapie-negative brain homogenates, and were tested for the accumulation of nascent PrP^Sc^ at passage three post-inoculation by immunoblotting and at passage four post-inoculation by ELISA. In all experiments, only scrapie-inoculated clone 439 accumulated PrP^Sc^ as determined by the detection of cell-associated PK-resistant PrP by immunoblotting (Fig 1A) and high β–sheet PrP by ELISA (Fig 1B, 439/X124 vs. 439/mock [*P* = 0.0044, paired *t*-test]). While there was a statistical significance between 438/X124 and 438/mock (*P* = 0.0065 for clone 438, paired *t*-test), the levels failed to be higher than the manufacturer’s cut-off value and no PrP^Sc^ was detected in the 438/X124 samples by routine immunoblotting (Fig 1A, left panel) or after concentration by PTA precipitation (S1 Fig). Finally, the statistical analysis of normalized units from ELISA experiments revealed a significant difference (*P* = 0.0048, unpaired *t*-test) between clones 438 and 439 after inoculation with scrapie-positive brain homogenates. Therefore, these results confirm the divergent prion permissiveness phenotypes between these ovine microglia clones and for brevity these clones will be henceforth referred to as highly permissive (clone 439) and poorly permissive (clone 438) microglia.

### PrP^C^ expression levels in ovine microglia clones

**Fig 2:**
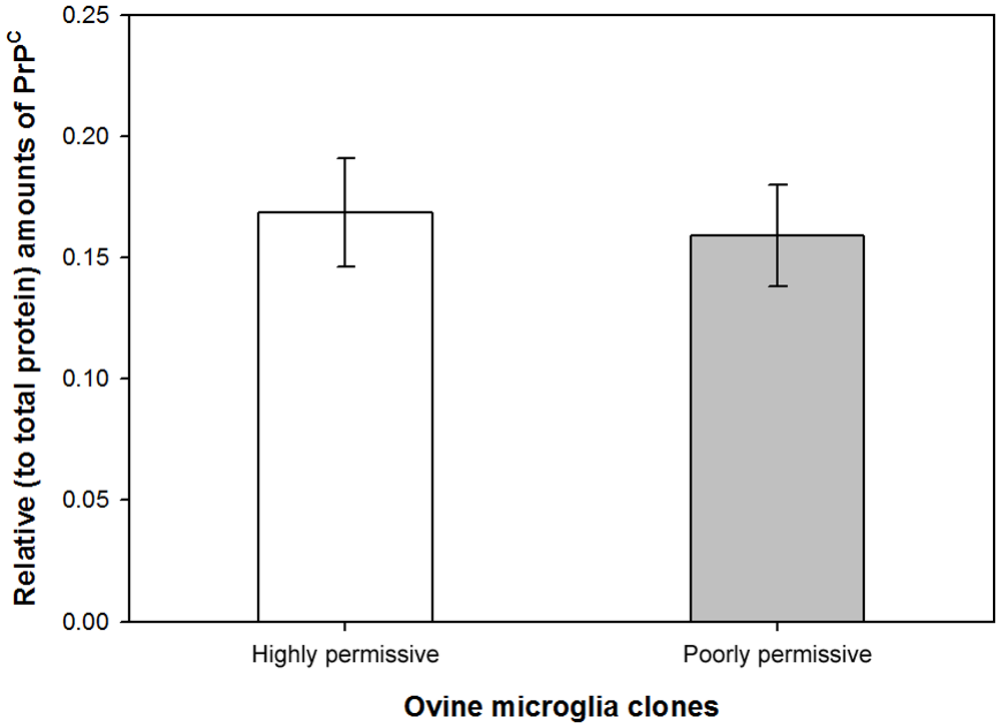
PrP^C^ expression in ovine microglia clones. Cell lysates of uninoculated cells were normalized to total protein and used to quantitatively analyze expression levels of endogenous PrP^C^ by ELISA. No significant difference in PrP^C^ levels was found (*P* = 0.573, paired *t*-test).

Expression of PrP^C^ in cultured cells is essential for the *ex vivo* propagation of PrP^Sc^ [5]. Thus, we investigated if the poorly permissive phenotype of ovine microglia clone 438 was due to markedly reduced expression of PrP^C^ as compared to highly permissive microglia clone 439. To determine levels of PrP^C^, lysates of uninoculated cells of each microglia clone were analyzed by ELISA using a standard curve. No difference in PrP^C^ levels was detected between the highly permissive and poorly permissive ovine microglia clones (*P* = 0.573, paired *t*-test, Fig 2). This finding suggests that this level of PrP^C^ is insufficient by itself to confer prion permissiveness in different ovine microglia clones.

### Comparative transcriptional analysis

**Fig 3:**
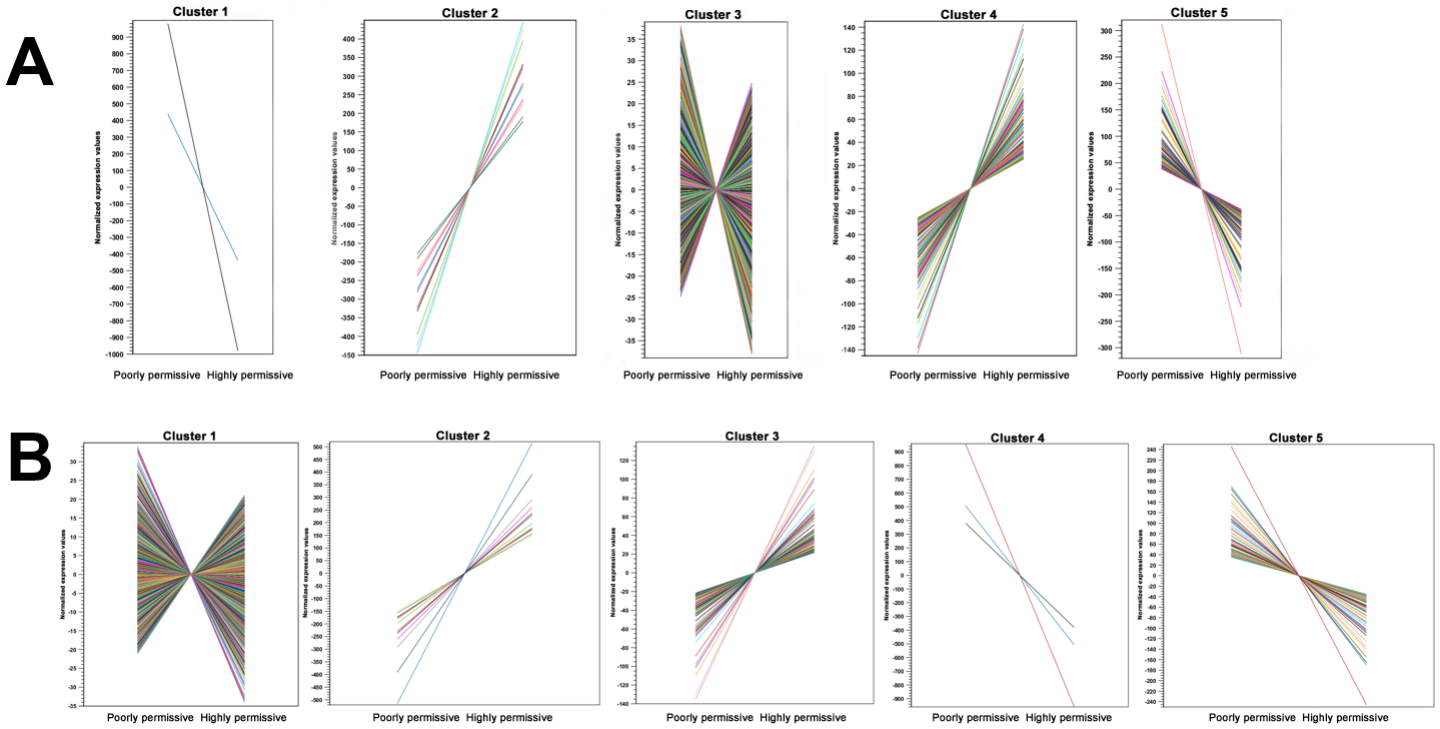
K-means clustering of genes with differential transcription in comparisons of highly permissive against poorly permissive microglia. The five clusters generated in the comparisons of mock-inoculated (A) and scrapie-inoculated (B) microglia clones are depicted. Each color line represents a single gene included in each cluster. The y—axis shows normalized expression values (RPKM), and microglia clones are on the x—axis.

RNA-Seq was used to compare the transcriptional profiles of highly permissive and poorly permissive ovine microglia clones after inoculation with either scrapie-positive or scrapie-negative (i.e., mock) brainstem homogenates. The number of reads per library ranged from 25.8 to 33.7 million, and the number of reads mapped to the *O*. *aries* genome [25] ranged from 17.2 to 22.1 million (Table 2). Mapped reads were normalized using RPKM values and normalized values were used for pair-wise comparisons between microglia clones under the same inoculation conditions (i.e., scrapie or mock). Fold changes in transcription were considered significant when Bonferroni-corrected *P* values (Baggerley’s test) were less than 0.05. K—means clustering was used to identify genes with similar transcriptional patterns. Most of the genes with altered transcription in the comparison of mock-inoculated microglia fell into clusters 3 and 4; these genes were only slightly up- or downregulated (Fig 3A). Clusters 1, 2, and 5 from the same comparison showed higher magnitude of transcription than clusters 3 and 4; thus, the genes within the former clusters were selected for further analysis. Likewise, based on the same criteria mentioned above, the genes with altered transcription in the scrapie-inoculated microglia comparison that fell into clusters 2, 4, and 5 were further analyzed (Fig 3B).

**Table 2:** Reads mapped to *O*. *aries* genome.

Experiment group (microglia clone/inoculum)		Total reads mapped to *O*. *aries* genome	% of reads mapped to *O*. *aries* genome
Highly permissive/Mock	30,150,424	19,341,429	64.15
	33,750,532	21,783,758	64.54
	29,777,612	17,288,403	58.06
Highly permissive/Scrapie	29,322,920	19,676,625	67.1
	29,179,988	17,751,264	60.83
	30,184,738	21,223,906	70.31
Poorly permissive/Mock	29,795,238	18,879,979	63.37
	28,227,946	19,430,079	68.71
	30,521,914	21,390,586	70.08
Poorly permissive/Scrapie	29,309,184	18,221,641	62.17
	33,028,046	22,097,393	66.9
	25,814,978	17,952,849	69.54

**Fig 4:**
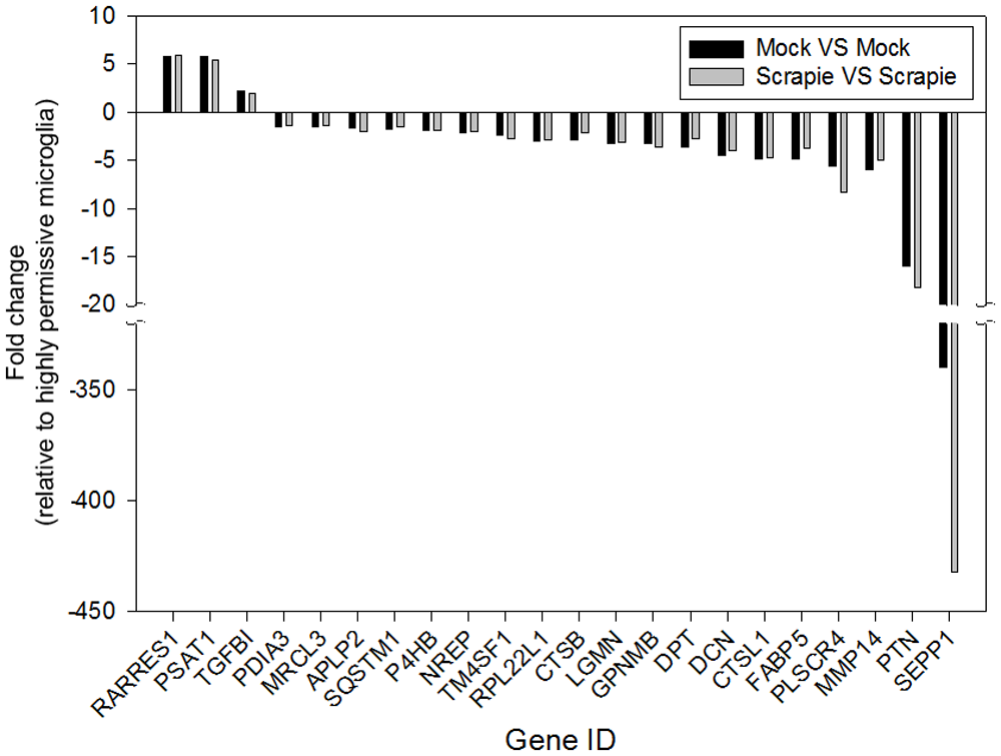
Transcript fold-change in highly permissive microglia compared to poorly permissive microglia. Transcriptional profiles of highly permissive and poorly permissive microglia clones under two experimental conditions (mock [black bars] or scrapie [gray bars] inoculation) were compared. All the genes with differential transcription in both comparisons (*P* < 0.05 [Baggerley’s test and Bonferroni correction]) and known biological function across three culture replicates are shown. Gene IDs are on the x—axis. The fold change in transcription relative to highly permissive microglia is on the y—axis. Thus, positive fold changes indicate upregulation in highly permissive microglia and negative fold changes indicate upregulation in poorly permissive microglia.

Treatment-matched pair-wise comparisons (i.e., highly permissive microglia/mock vs. poorly permissive microglia/mock and highly permissive microglia/scrapie vs. poorly permissive microglia/scrapie) revealed 82 genes with altered transcription in the comparison of mock-inoculated microglia and 40 genes in the comparison of scrapie-inoculated microglia (S1 File). Fifty-seven genes with altered transcription from the comparison of mock-inoculated microglia and 32 from the scrapie-inoculated microglia comparison had known biological function (thus, there are 25 and 8 hypothetical loci identified, respectively [Tables 3 and 4]). Of these, 7/57 and 6/32 genes were upregulated in highly permissive microglia, and 50/57 and 26/32 were upregulated in poorly permissive microglia. The transcriptional status of 22 genes with known biological function (*APLP2*, *CTSB*, *CTSL1*, *DCN*, *DPT*, *FABP5*, *GPNMB*, *LGMN*, *MMP14*, *NREP*, *MRCL3*, *P4HB*, *PDIA3*, *PLSCR4*, *PSAT1*, *PTN*, *RARRES1*, *RPL22L1*, *SEPP1*, *SQSTM1*, *TGFBI*, and *TM4SF1*) was consistently altered in both pair-wise comparisons (Fig 4). The fold change in transcription of these genes varied from 1.26 to 432.14, with *SEPP1*, having the most dramatic change in transcription (i.e., 339.85 fold change in the mock-inoculated comparison and 432.14 fold change in the scrapie-inoculated comparison). Treatment-mismatched comparisons (i.e., mock vs. scrapie and scrapie vs. mock) revealed similar results (S2 Fig and S1 File) as those mentioned above with the addition of genes *ITM2B* (up-regulated in highly permissive microglia), and *CRLS1* and *RPS20* (up-regulated in poorly permissive microglia). For the treatment-matched pair-wise comparisons, the 33 hypothetical genes were analyzed for conserved domains (Tables 3 and 4), but only seven were consistently altered in both comparisons. Hypothetical genes from the treatment-mismatched pair-wise comparisons were not further analyzed.

**Table 3:** Genes encoding for hypothetical proteins with altered transcription in mock-inoculated microglia clones.

Feature symbol	FC	Identity	Conserved domains on	E-value
LOC101104567^¶^	9.36	Envelope glycoprotein-like	Env polyprotein*	2.18E-110
LOC101122294	3.67	Membrane cofactor protein-like	Complement control modules	6.90E-12
LOC101116132	-1.46	40S ribosomal protein S3a-like	Ribosomal S3Ae family	1.21E-100
LOC101106384	-1.53	Thymosin beta-4-like	Thymosin beta-4 family	4.19E-14
LOC101110467^¶^	-1.66	Transmembrane protein 45A-like	Family of unknown function (DUF716)	3.44E-41
LOC101103097	-1.68	Translationally-controlled tumor protein-like	Translationally controlled tumor protein	1.23E-60
LOC101104079	-1.81	Cystatin C	Cystatin-like domain	8.98E-36
LOC101109246	-1.82	40S ribosomal protein S4-like	KOW motif of ribosomal protein S4	2.10E-28
LOC101105484^¶^	-2.11	Ferritin heavy chain-like	Eukaryotic ferritins	1.10E-93
LOC443512^¶^	-2.16	Collagen I pro-alpha 2 chain precursor	Fibrillar collagens C-terminal domain	1.18E-134
LOC100037666	-2.20	Ribosomal protein S11	40s ribosomal protein S11	5.93E-91
LOC100037669	-2.23	Niemann-Pick disease type C2	Niemann-Pick type C2	7.10E-54
LOC101104961	-2.27	40S ribosomal protein S24-like	40S ribosomal protein S24	1.52E-43
LOC101104661	-2.34	Growth-regulated alpha protein-like	Chemokin_CXC	1.21E-24
LOC101103639	-2.48	Uncharacterized LOC101103639	None	None
LOC100037665	-2.52	Ribosomal protein s6	Ribosomal protein S6e	2.60E-101
LOC780524	-2.54	Ribosomal protein S2	Ribosomal protein S5, N-terminal domain	3.21E-30
LOC100101231^¶^	-2.56	Collagen type III alpha 1	Fibrillar collagens C-terminal domain	7.92E-138
LOC101122112	-2.68	60S ribosomal protein L36a-like	60S ribosomal protein L36a	1.93E-95
LOC100037664	-2.76	Ribosomal protein L35a	Ribosomal protein L35Ae	3.17E-53
LOC101112245	-2.77	Adenosylhomocysteinase-like	S-adenosylhomocysteine hydrolase	0E+00
LOC100037667	-3.29	Ribosomal protein S12	Ribosomal protein L7A3/L30e/S12e/Gadd45 family	8.51E-30
LOC101102096	-3.81	Laminin receptor 1 pseudogene	40S ribosomal protein SA*	9.07E-125
LOC101108131^¶^	-3.89	Complement C3-like	Proteins similar to C3, C4, C5 of vertebrate complement	1.90E-94
LOC101102861^¶^	-5.31	Phospholipid scramblase 2-like	Scramblase	1.42E-122

FC: fold change in transcription (relative to highly permissive microglia).

*: Protein sequence manually generated prior to identification of conserved domains.

^¶^: Genes with altered transcription in both treatment-matched comparisons.

**Table 4:** Genes encoding for hypothetical proteins with altered transcription in scrapie-inoculated microglia clones.

Gene symbol	FC	Identity	Conserved domains	E-value
LOC101104567^¶^	8.31	Envelope glycoprotein-like	Env polyprotein*	2.18E-110
LOC101105484^¶^	-1.55	Ferritin heavy chain-like	Eukaryotic ferritins	1.10E-93
LOC101110467^¶^	-1.68	Transmembrane protein 45A-like	Family of unknown function (DUF716)	3.44E-41
LOC101103238	-2.06	Uncharacterized LOC101103238	Chemokine_CXC	2.09E-23
LOC443512^¶^	-2.27	Collagen I pro-alpha 2 chain precursor	Fibrillar collagens C-terminal domain	1.18E-134
LOC101108131^¶^	-3.08	Complement C3-like	Proteins similar to C3, C4, C5 of vertebrate complement	1.90E-94
LOC100101231^¶^	-3.13	Collagen type III alpha 1	Fibrillar collagens C-terminal domain	7.92E-138
LOC101102861^¶^	-6.29	Phospholipid scramblase 2-like	Scramblase	1.42E-122

FC: Fold change in transcription (relative to highly permissive microglia).

*: Protein sequence manually generated prior to identification of conserved domains.

^¶^: Genes with altered transcription in both treatment-matched comparisons.

GSEA identified 59 altered biological pathways in the comparison of mock-inoculated clones and 46 altered biological pathways in the comparison of scrapie-inoculated clones (*P <* 0.01, S2 File). Of these, twenty-one pathways were similarly altered in both comparisons (Tables 5 and 6), and the proteolysis, positive regulation of cell migration, chromosome segregation, translation, and mitosis pathways had the lowest scores. Proteolysis, translation, and mitosis consistently included the highest number of features, suggesting significant alteration of these pathways. Genes of the *Ovis aries* genome assigned to biological pathways are listed in S4 File.

**Table 5:** Significantly altered biological pathways in highly permissive vs. poorly permissive microglia comparison after mock inoculation.

Category	Description	Size	Test statistic	Lower tail	Upper tail
6412	translation (GO_REF:0000002 [IEA]q InterPro:IPR000039|InterPro:IPR021132)	109	-42.079	0	1
30335	positive regulation of cell migration (GO_REF:0000024 [ISS] UniProtKB:Q8N4T4)	76	-16.3188	0.0003	0.9997
7059	chromosome segregation (GO_REF:0000019 [IEA] Ensembl:ENSP00000362702)	34	-17.8086	0.0004	0.9996
6508	proteolysis (GO_REF:0000003 [IEA] EC:3.4.19.9)	372	-13.713	0.0006	0.9994
71285	cellular response to lithium ion (GO_REF:0000019 [IEA] Ensembl:ENSMUSP00000020974)	11	-20.2306	0.0009	0.9991
70830	tight junction assembly (GO_REF:0000019 [IEA] Ensembl:ENSP00000345731)	13	-18.1835	0.0015	0.9985
6270	DNA replication initiation (GO_REF:0000002 [IEA] InterPro:IPR003874)	12	-16.9102	0.0016	0.9984
7067	mitosis (GO_REF:0000019 [IEA] Ensembl:ENSP00000297596)	97	-13.8772	0.0016	0.9984
60766	negative regulation of androgen receptor signaling pathway (GO_REF:0000019 [IEA] Ensembl:ENSP00000362649)	12	-17.9763	0.0018	0.9982
90102	cochlea development (GO_REF:0000019 [IEA] Ensembl:ENSMUSP00000077492)	10	-16.9598	0.0025	0.9975
22408	negative regulation of cell-cell adhesion (GO_REF:0000019 [IEA] Ensembl:ENSP00000354040)	10	-16.8262	0.0031	0.9969
3382	epithelial cell morphogenesis (GO_REF:0000019 [IEA] Ensembl:ENSMUSP00000128056)	10	-15.6154	0.0045	0.9955
51301	cell division (GO_REF:0000037 [IEA] UniProtKB-KW:KW-0132)	44	-12.6405	0.0046	0.9954
71560	cellular response to transforming growth factor beta stimulus (GO_REF:0000019 [IEA] Ensembl:ENSP00000457230)	21	-13.6831	0.0046	0.9954
7076	mitotic chromosome condensation (GO_REF:0000002 [IEA] InterPro:IPR027120)	11	-13.4107	0.0076	0.9924
21766	hippocampus development (GO_REF:0000019 [IEA] Ensembl:ENSMUSP00000019911)	29	12.99072	0.9939	0.0061
2088	lens development in camera-type eye (GO_REF:0000019 [IEA] Ensembl:ENSMUSP00000087870)	16	12.89431	0.9947	0.0053
48013	ephrin receptor signaling pathway (GO_REF:0000019 [IEA] Ensembl:ENSP00000332118)	31	14.16371	0.9951	0.0049
42733	embryonic digit morphogenesis (GO_REF:0000019 [IEA] Ensembl:ENSMUSP00000019911)	46	13.21356	0.9952	0.0048
16358	dendrite development (GO_REF:0000019 [IEA] Ensembl:ENSMUSP00000019911)	29	15.72994	0.9976	0.0024
42384	cilium assembly (GO_REF:0000019 [IEA] Ensembl:ENSP00000424757)	68	16.71427	0.9981	0.0019

Lower and Upper tail values show the mass in the permutation based p-value distribution below or above the value of the test statistic. *P* values represented as 0 are less than 10^-16^.

**Table 6:** Significantly altered biological pathways in highly permissive vs. poorly permissive microglia comparison after scrapie inoculation.

Category	Pathway description	Size	Test statistic	Lower tail	Upper tail
6508	proteolysis (GO_REF:0000003 [IEA] EC:3.4.19.9)	372	-17.1577	0	1
30335	positive regulation of cell migration (GO_REF:0000024 [ISS] UniProtKB:Q8N4T4)	76	-21.8761	0	1
7067	mitosis (GO_REF:0000019 [IEA] Ensembl:ENSP00000297596)	97	-22.4604	0.0001	0.9999
7059	chromosome segregation (GO_REF:0000019 [IEA] Ensembl:ENSP00000362702)	34	-21.7366	0.0002	0.9998
70830	tight junction assembly (GO_REF:0000019 [IEA] Ensembl:ENSP00000345731)	13	-18.3619	0.0008	0.9992
7076	mitotic chromosome condensation (GO_REF:0000002 [IEA] InterPro:IPR027120)	11	-18.4568	0.0014	0.9986
71285	cellular response to lithium ion (GO_REF:0000019 [IEA] Ensembl:ENSMUSP00000020974)	11	-19.3928	0.0016	0.9984
6270	DNA replication initiation (GO_REF:0000002 [IEA] InterPro:IPR003874)	12	-15.5962	0.0039	0.9961
90102	cochlea development (GO_REF:0000019 [IEA] Ensembl:ENSMUSP00000077492)	10	-13.4579	0.0046	0.9954
6412	translation (GO_REF:0000002 [IEA] InterPro:IPR000039|InterPro:IPR021132)	109	-12.6216	0.0048	0.9952
22408	negative regulation of cell-cell adhesion (GO_REF:0000019 [IEA] Ensembl:ENSP00000354040)	10	-13.3994	0.0049	0.9951
60766	negative regulation of androgen receptor signaling pathway (GO_REF:0000019 [IEA] Ensembl:ENSP00000362649)	12	-13.9031	0.0052	0.9948
71560	cellular response to transforming growth factor beta stimulus (GO_REF:0000019 [IEA] Ensembl:ENSP00000457230)	21	-12.6096	0.0062	0.9938
3382	epithelial cell morphogenesis (GO_REF:0000019 [IEA] Ensembl:ENSMUSP00000128056)	10	-13.3184	0.0066	0.9934
51301	cell division (GO_REF:0000037 [IEA] UniProtKB-KW:KW-0132)	44	-11.682	0.0083	0.9917
2088	lens development in camera-type eye (GO_REF:0000019 [IEA] Ensembl:ENSMUSP00000087870)	16	9.050416	0.9912	0.0088
42733	embryonic digit morphogenesis (GO_REF:0000019 [IEA] Ensembl:ENSMUSP00000019911)	46	8.110665	0.992	0.008
48013	ephrin receptor signaling pathway (GO_REF:0000019 [IEA] Ensembl:ENSP00000332118)	31	8.717496	0.9922	0.0078
42384	cilium assembly (GO_REF:0000019 [IEA] Ensembl:ENSP00000424757)	68	7.913188	0.9932	0.0068
21766	hippocampus development (GO_REF:0000019 [IEA] Ensembl:ENSMUSP00000019911)	29	8.947898	0.9946	0.0054
16358	dendrite development (GO_REF:0000019 [IEA] Ensembl:ENSMUSP00000019911)	29	9.441165	0.9947	0.0053

Lower and Upper tail values show the mass in the permutation based p-value distribution below or above the value of the test statistic. *P* values represented as 0 are less than 10^-16^.

### Validation of RNA-Seq results by RT-qPCR

**Fig 5:**
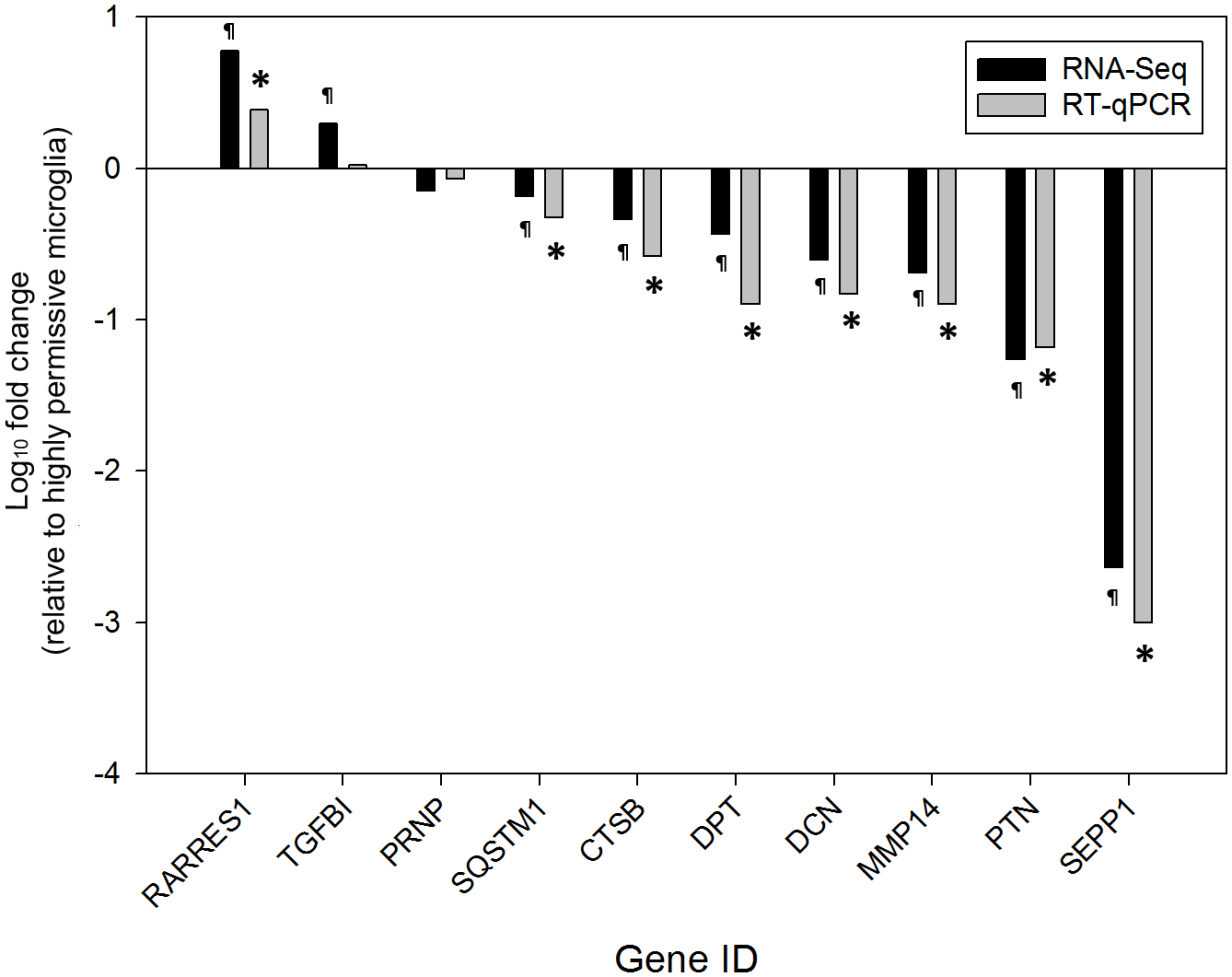
Validation of RNA-Seq results by RT-qPCR. The fold changes in transcription of 10 genes across nine culture replicates of ovine microglia clones inoculated with scrapie prions are shown. Black bars represent RNA-Seq results and gray bars represent RT-qPCR results. RNA-Seq results were confirmed by RT-qPCR in 9 of 10 cases. ¶: *P* < 0.05. *: *P* < 0.0001. Gene IDs are on the x—axis. The log_10_ fold change in transcription relative to highly permissive microglia is on the y—axis. Thus, positive fold changes indicate upregulation in highly permissive microglia and negative fold changes indicate upregulation in poorly permissive microglia.

To confirm the RNA-Seq results, the transcriptional status of nine genes (*CTSB*, *DCN*, *DPT*, *MMP14*, *PTN*, *RARRES1*, *SEPP1*, *SQSTM1*, and *TGFBI*) with different transcriptional patterns and one gene (*PRNP*) without change in transcription (according to the RNA-Seq data) was analyzed by RT-qPCR. Only the experimental groups inoculated with natural scrapie prions were selected for this analysis. The transcriptional status of nine genes (*CTSB*, *DCN*, *DPT*, *MMP14*, *PTN*, *SEPP1*, *PRNP*, and *SQSTM*) obtained from the RNA-Seq experiment was confirmed by RT-qPCR (Fig 5). Upregulation of *TGFBI* in highly permissive microglia was not confirmed.

### Identification of genes enriched in ramified and amoeboid microglia

Microglia derive from circulating monocytes that originate from the bone marrow; during post-natal life, these cells migrate into the brain and two morphologically different populations are recognized: amoeboid and ramified microglia [39]. Given that the cells used in this study had been only characterized as monocyte-derived cells by expression of CD14 [7], we sought to determine if these microglia clones were more consistent with either ramified or amoeboid microglia by identifying the presence/absence of expression of genes enriched depending on the microglia phenotype. Due to the lack of sheep-specific amoeboid and ramified microglia gene database, a published gene database from rat microglia was used as reference [37]. Both groups of microglia clones (mock-inoculated and scrapie-inoculated, three replicates each) were considered for this analysis. Genes were considered expressed when their RPKM average values were ≥1 [38]. IDs of genes analyzed are all listed in S3 File. Out of 43 genes enriched in rat amoeboid microglia, 38 genes were mappable to the ovine microglia RNA-Seq database. Thirty-one (82%) mappable genes enriched in neonatal amoeboid microglia were expressed in poorly permissive microglia and had an average RPKM of 50.9 (mock-inoculated) and 51.9 (scrapie-scrapie-inoculated); and 29 (76%) were expressed in highly permissive microglia with an average RPKM of 51.4 (mock-inoculated) and 56.2 (scrapie-inoculated) (Table 7 and S3 File). On the other hand, out of 42 genes enriched in rat ramified microglia, 39 genes were mappable to the ovine microglia RNA-Seq database. Of these, only 15 (38%) were expressed in poorly permissive microglia and had an average RPKM of 7.3 (mock-inoculated) and 6.3 (scrapie inoculated); and 14 (36%) were in highly permissive microglia with an average RPKM of 5.9 (mock-inoculated) and 6.2 (scrapie-inoculated) (Table 7 and S3 File). These findings suggest that the genotype of the ovine microglia clones used in this study is more consistent with neonatal amoeboid microglia rather than ramified microglia.

**Table 7:** Enriched genes of rat amoeboid microglia present in ovine microglia.

Gene group	Poorly permissive microglia		Highly permissive microglia	
	Mock	Scrapie	Mock	Scrapie
Mappable amoeboid genes (n = 38)	31	31	29	29
expressed in microglia (%)	(82%)	(82%)	(76%)	(76%)
Mappable ramified genes (n = 39)	15	14	14	14
expressed in microglia	(38%)	(36%)	(36%)	(36%)

## Discussion

Identification of cellular factors associated with prion conversion and degradation would greatly improve the understanding of TSEs pathogenesis and enable investigation of therapeutic interventions. It is known that PrP^C^ is essential for replication of PrP^D^ in animal models [40] and cultured cells [5]; however, the presence of PrP^C^ does not guarantee prion conversion, highlighting the requirement for additional cell-associated factors. In this study, an immortalized microglia *ex vivo* system, derived from a natural TSE host, was used to identify potential cellular factors associated with relative prion permissiveness and resistance. This was accomplished by using RNA-Seq to compare the global transcriptional profiles of two clonal populations of microglia with differential prion permissiveness.

When comparing between permissibility phenotypes, transcriptional analysis identified 40 and 82 genes with altered transcription in two pair-wise comparisons, of which only 32 and 57 genes have known biological function. Twenty-two genes with known biological functions were consistently altered in both comparisons between highly permissive and poorly permissive microglia. The relative paucity of differential transcription indicates the high transcriptomic similarity between these two clonal populations of ovine microglia. Furthermore, the transcriptome suggests that the ovine microglia cells used in this study are more likely to be amoeboid microglia. This finding is expected as amoeboid microglia predominate in developing (fetal) brains, from which these microglia were isolated.

In regards to transcriptomic differences between highly permissive and poorly permissive clones, the fold change in transcription of these genes varied from 1.26- to 432.14-fold. Thirty-three genes encoding for hypothetical proteins were identified as differentially regulated; however, the significance of these is more tenuous based on the lack of definitive biological function. When comparing between inoculation statuses, most of the transcriptional differences between highly permissive and poorly permissive microglia appeared not to be induced by prion inoculation. This indicates that altered transcription of most of these genes is likely a pre-existing condition in these microglia clones.

Most of the genes with altered transcription were upregulated in poorly permissive microglia. *SEPP1* was the gene with the most dramatic fold change in transcription in this study; it encodes for selenoprotein P, an extracellular selenium transporter glycoprotein that contains most of the selenium in plasma [41]. Selenoprotein P co-localizes with amyloid-β plaques and neurofibrillary tangles in individuals with Alzheimer’s disease [42] and inhibits aggregation and neurotoxicity of amyloid-β in mouse neuroblastoma cells [43]. Similarly, in poorly permissive ovine microglia, aggregation of PrP^Sc^ may be inhibited by selenoprotein P.

Multiple genes encoding for enzymes involved in proteolysis were upregulated in poorly permissive microglia. Of these, *CTSB* and *CTSL1* encode for the cysteine proteases cathepsin B and cathepsin L, correspondingly. These cathepsins are located within lysosomal compartments and plasma membrane [44], subcellular locations in which prion conversion is thought to occur [45–47]. In murine neuronal (GT1-1) [48] and bone marrow-derived dendritic cells [19], cathepsins B and L partially degrade prions. Thus, in ovine microglia, it is possible that cathepsins B and L degrade internalized and cell membrane-associated scrapie prions. The potential effect of these proteases in poorly permissive microglia appears to be PrP^Sc^-specific as no quantitative (see Fig 3) and qualitative [7] differences are found in PrP^C^ between the two ovine microglia clones.

In neuroblastoma cells, the upregulation of matrix metalloproteinases (MMPs) 2 and 9 results in decreased deposition of PrP^C^ at the extracellular matrix and resistance to prion infection [13]. Relatedly in this study, *MMP14*, an important activator of *MMP2* [49], was consistently upregulated in poorly permissive microglia. Furthermore, in poorly permissive cells, *MMP2* and *FN1* were found to be upregulated, but only when these cells were inoculated with scrapie prions (see S1 File). These findings suggest that expression of *FN1* and *MMP2* is a response of poorly permissive microglia to inoculation with scrapie prions and reinforce the proposal by Marbiah *et al* [13] that *FN1* activates expression of MMPs. Also, an alternative pathway of MMP activation in ovine microglia may involve *HTRA1*, which was upregulated in poorly permissive cells in two comparisons (see S1 File). *HTRA1* encodes for a serine protease that targets extracellular matrix components (including fibronectin) and whose degradation products increase expression of MMPs [50]. Studies to further confirm and elucidate the potential role of MMPs in cellular permissibility to prions are ongoing.

*SQSTM1* encodes for sequestosome 1, a protein required for the degradation of polyubiquitin-containing bodies that has been co-localized with intraneuronal ubiquitinated protein aggregates in individuals with protein misfolding diseases [51–53]. In mouse neuronal and microglia culture systems, *SQSTM1* is overexpressed after inoculation with mouse-derived prions and is associated with degradation of PrP^Sc^ [54]. Our findings regarding *SQSTM1* transcription are consistent with the latter studies and indicate that sequestosome 1 may contribute to degradation of scrapie prions in cultured cells of sheep, a natural TSE host. The proteins encoded by *APLP2*, *PTN*, *DCN*, *GPNMB*, *P4HB*, and *PDIA3*, have been associated with either protein misfolding diseases or TSEs [8, 55–59] but their specific role in prion protein degradation has not been described. Also, to the authors’ knowledge, the genes *LGMN*, *SERPINH1*, *DPT*, *NREP*, *PLSCR4*, *FABP5*, *RPL22L1*, *TM4SF1*, and *MRCL3* have not been associated with either TSEs or protein misfolding disorders and their speculative role in resistance to prion infection is unclear at the moment. Genes encoding for multiple characterized and putative ribosomal proteins were upregulated in poorly permissive microglia after mock inoculation, but remained without significant change in transcription after scrapie infection as compared to the corresponding highly permissive microglia. The reason and significance for this difference is unclear.

Six to seven genes were upregulated in highly permissive microglia and only *RARRES1*, *PSAT1*, and *TGFBI* were consistently upregulated across pair-wise comparisons. The gene *TGFBI* encodes for the transforming growth factor beta-induced protein, which contributes to cell-collagen interaction and has been linked to protein aggregates in individuals with corneal dystrophy [60]. Moreover, mutants of *TGFBI* induce *ex vivo* aggregation of amyloid–β [61]. Thus, the transforming growth factor beta-induced protein may favor accumulation of scrapie prions by increasing aggregation of PrP^Sc^ in highly permissive microglia; however, RT-qPCR failed to verify altered transcript levels of *TGFBI*. *RARRES1* encodes for the membrane-associated retinoic acid receptor responder membrane protein. While there is no previous evidence of this specific protein contributing to prion disease pathogenesis, retinoic acid treatment of N2a cells did increase their prion permissibility [13].

As mentioned above, our stringent algorithm and previous studies [13] have demonstrated a potential role for extracellular matrical proteins in prion permissibility, such as *FN1* and *MMP2*, a modulator of the matrix. Furthermore, eleven genes (*LOC101103238*, *ATP5A1*, *CYTB*, *FN1*, *FSTL1*, *HSP90B1*, *ITM2B*, *LMO4*, *MDD2*, *ND5*, and *VWA5A*) are differentially regulated following prion inoculation (S1 File), but are not included as differentially expressed following mock inoculation. The gene *LOC101103238* (recently named *CXCL5*) and follistatin (*FST*) have been shown to be upregulated in mice inoculated with 22L prions prior to the development of clinical signs [62] and in prion susceptible neuroblastoma cells [13], correspondingly. Likewise, *ND5* and *VWA5A* have been found to be upregulated in patients with Creutzfeldt-Jakob disease [63] and sheep infected with scrapie [8], respectively. However, the specific role of the latter genes in prion diseases and/or permissiveness to prion infection has not been characterized.

GSEA identified several altered biological pathways with proteolysis, translation, and mitosis being the most consistently affected in both comparisons. The results of this analysis are consistent with two trends observed in poorly permissive microglia. The first corresponds to the proteolysis pathway, as discussed above. The second trend includes the alteration of translation pathway. Many genes encoding for ribosomal proteins were upregulated in poorly permissive microglia; however, the speculative impact of this pathway and these genes in prion infection resistance is unclear.

## Conclusions

The global transcriptional profiles of two ovine microglia clones with differential scrapie prion permissiveness were compared using RNA-Seq, resulting in identification of 22 genes with consistently altered transcription and known biological function. The transcription of several other genes was altered but their difference in transcription was inconsistent and, in some cases, the biological function of such genes was unknown. Most of the genes with altered transcription were upregulated in poorly permissive microglia. The proteins encoded by many of these genes have known activity that may contribute to prion resistance (*CTSB*, *CTSL1*, *SEPP1*, and *SQSTM1*), possibly by degradation or neutralization of prions. The role in resistance to prion infection of other genes (*APLP2*, *DCN*, *DPT*, *FABP5*, *GPNMB*, *MRCL3*, *P4HB*, *PDIA3*, *PLSCR4*, *PTN*, *RPL22L1*, and *TM4SF1*) remains unclear. Only three genes, *RARRES1*, *PSAT1*, and *TGFBI*, were consistently upregulated in highly permissive microglia. Overall, the transcriptomic similarity of these clones and the overlapping results (e.g., *MMP14* and *FN1*) with previous studies support the relevance of these findings, providing new insights in the cellular pathophysiology of TSEs and new candidate genes for therapeutic targets and markers of prion resistance and susceptibility.

## Supporting Information

S1 Fig PTA precipitation of PK-resistant PrP from microglia lysates. At passage 3 post-inoculation, cell lysates were collected, treated with PK, and incubated with PTA to increase sensitivity of immunoblotting. PK-resistant PrP was precipitated with PTA only from cells of clone 439. The results of three independent culture replicates inoculated with scrapie-positive brainstem homogenates (PrP^Sc^ +, lanes 1–3 and 5–7) and one with scrapie-negative inoculum (PrP^Sc^–, lanes 4 and 8) of each microglia clone are shown, and are representative of three experiments.
(TIFF)

S2 Fig Transcript fold-change in highly permissive microglia compared to poorly permissive microglia under different inoculation conditions. Transcriptional profiles of highly permissive and poorly permissive microglia clones under two different inoculation conditions were compared (i.e., mock VS scrapie and scrapie VS mock). Genes with differential transcription in both comparisons (*P* < 0.05 [Baggerley’s test and Bonferroni correction]) and known biological function across three culture replicates are shown. Gene IDs are on the x—axis and the fold change in transcription relative to highly permissive microglia is on the y—axis. Positive fold changes indicate up-regulation in highly permissive microglia and negative fold changes indicate up-regulation in poorly permissive microglia.
(TIF)

S1 File Comparative transcriptional profiling of highly permissive versus poorly permissive microglia. (XLSX)

S2 File GSEA on transcriptional profiling of highly permissive and poorly permissive ovine microglia. (XLSX)

S3 File Assessment of ovine microglia for the presence/absence of amoeboid and ramified microglia. (XLSX)

S4 File Genes in *Ovis aries* genome assigned to biological pathways. (XLSX)
