# Supplementary figures and images for: Transcriptomic Determinants of Scrapie Prion Propagation in Cultured Ovine Microglia
Source: PLoS One. 2016 Jan 25;11(1):e0147727. doi: 10.1371/journal.pone.0147727 (PMC4726464; doi:10.1371/journal.pone.0147727)

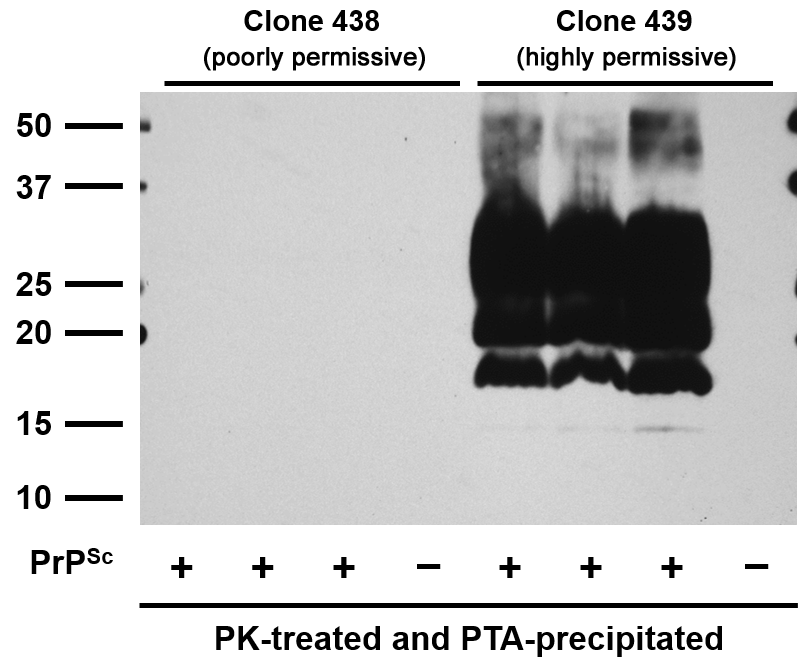

Supplement: S1 Fig — At passage 3 post-inoculation, cell lysates were collected, treated with PK, and incubated with PTA to increase sensitivity of immunoblotting. PK-resistant PrP was precipitated with PTA only from cells of clone 439. The results of three independent culture replicates inoculated with scrapie-positive brainstem homogenates (PrPSc +, lanes 1–3 and 5–7) and one with scrapie-negative inoculum (PrPSc–, lanes 4 and 8) of each microglia clone are shown, and are representative of three experiments. (TIFF) [file pone.0147727.s001.tiff]

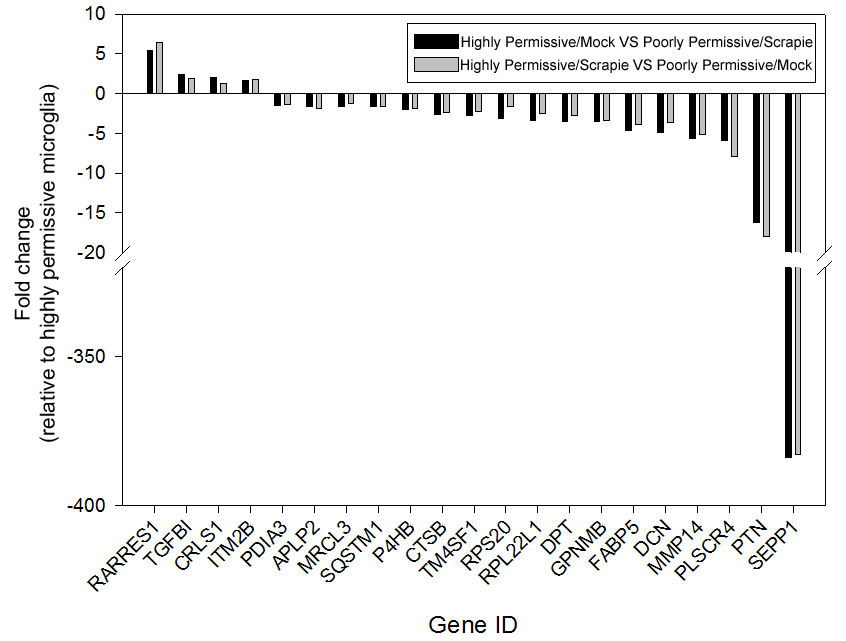

Supplement: S2 Fig — Transcriptional profiles of highly permissive and poorly permissive microglia clones under two different inoculation conditions were compared (i.e., mock VS scrapie and scrapie VS mock). Genes with differential transcription in both comparisons (P < 0.05 [Baggerley’s test and Bonferroni correction]) and known biological function across three culture replicates are shown. Gene IDs are on the x—axis and the fold change in transcription relative to highly permissive microglia is on the y—axis. Positive fold changes indicate up-regulation in highly permissive microglia and negative fold changes indicate up-regulation in poorly permissive microglia. (TIF) [file pone.0147727.s002.tif]
